# A Case Report of Severe Coagulopathy in Antenatal COVID-19

**DOI:** 10.1155/2022/7777445

**Published:** 2022-08-26

**Authors:** Anna Sand, Roza Chaireti, Maria Sennström, Karin Pettersson, Maria Magnusson

**Affiliations:** ^1^Department of Women's and Children's Health, Karolinska Institutet, Stockholm, Sweden; ^2^Department of Obstetrics and Gynaecology, Karolinska University Hospital, Stockholm, Sweden; ^3^Department of Molecular Medicine and Surgery, Karolinska Institutet, Stockholm, Sweden; ^4^Coagulation Unit, Department of Hematology, Karolinska University Hospital, Solna, Stockholm, Sweden; ^5^Department of Clinical Science, Intervention and Technology, Karolinska Institutet, Stockholm, Sweden

## Abstract

Pregnancy is a naturally occurring hypercoagulable state, and COVID-19 can cause profound changes in the coagulation system associated with thromboinflammation. We report a case of a pregnant woman with moderate symptoms of COVID-19 and a severe coagulopathy with unexpected low levels of fibrinogen and factor VIII as well as atypical thrombelastometry results. She developed a severe placental dysfunction with intrauterine fetal distress and perinatal death. The case did not fulfil the criteria for preeclampsia or sepsis, and the adverse outcome was assessed as a direct effect of the COVID-19 infection with placental insufficiency, despite absence of serious maternal pulmonary symptoms. Atypical persistent coagulopathy may serve as an important marker of a serious obstetrical situation in COVID-19.

## 1. Introduction

Pregnancy is a naturally occurring hypercoagulable state characterized by increased fibrinogen and D-dimer values and shorter activated partial thromboplastin time (aPTT) and prothrombin time (PT). The development of coagulopathy in pregnancy is often associated with preeclampsia, which is defined as hypertension accompanied by proteinuria, other maternal organ dysfunction or uteroplacental dysfunction at or after 20 weeks of gestation [1]. The HELLP syndrome is a serious complication of preeclampsia with coagulopathy characterized by hemolysis, elevated liver enzymes, and low platelet count. HELLP syndrome occurs in 0.5 to 0.9% of all pregnancies and in 10–20% of cases with severe preeclampsia [2].

COVID-19 can cause profound changes in the coagulation system, typically involving increased levels of D-dimer, fibrinogen, factor VIII (FVIII), and von Willebrand factor (vWF) and mild thrombocytopenia or thrombocytosis. The COVID-19-associated coagulopathy is a prothrombotic condition associated with disease severity and mortality [3, 4].

Here, we describe the case of a pregnant patient with COVID-19 and atypical coagulopathy.

## 2. Case Report

The patient was a 33-year-old pregnant woman of Northeast African origin, previously healthy and with a normal body mass index (BMI) of 21. She was gravida four, para two with one legal abortion, one previous late premature delivery, and one normal delivery in term pregnancy. She had no past obstetric history of preeclampsia or HELLP syndrome.

She was spontaneously pregnant with dichorionic diamniotic twins, and therefore, prophylaxis against preeclampsia with 75 mg acetylsalicylic acid was initiated at pregnancy week 14. At the routine ultrasound examination at gestational week 20 (19+3), twin number one (T1) was diagnosed with a unilateral cleft lip palate. The rest of the examination of both twins was unremarkable, and invasive fetal genetic testing was not performed.

The patient had not been vaccinated against COVID-19. In August 2021, ten days before presentation, she fell ill with muscular pain and a low-grade fever, without respiratory symptoms. She tested positive for the delta variant of COVID-19 three days later.

She presented with moderate lower abdominal pain and generally tense muscles at gestational week 25+0 and was admitted to the antenatal ward. Her blood pressure was 116/65 and body temperature 36.7°C. Chest CT-scan and abdominal and cardiac ultrasound of the mother were unremarkable, and the cardiotocographic registration trace (CTG) of the twins was normal. The platelet count, urate, fibrinogen levels, and aPTT were normal, she had moderate proteinuria, and the levels of D-dimer and liver enzymes (AST and ALT) were elevated at admission (Table 1). Thromboprophylaxis with dalteparin 5000 U/mL two times daily was initiated.

Day two after admission, she had a short episode of fever and the myalgia worsened. The fibrinogen was rapidly declining (0.7 g/L), whereas aPTT (>60 s) and lactate dehydrogenase (>10 *μ*kat/L) were rising, and the D-dimer was extremely elevated (>35 mg/L). Thromboelastography (ROTEM) showed in general prolonged clotting time (CT), clot formation time (CFT), and low maximum clot firmness (MCF). CT was especially pronounced in INTEM (CT 407 sec.), which could be partly due to a heparin effect since HEPTEM CT was only moderately elevated (CT 295 sec.). The low MCF was in concordance with hypofibrinogenemia. Further laboratory assays showed elevated level of vWF and unexpectedly low level of FVIII (0.37-0.39 kIU/L) (Table 1).

The viral load of COVID-19 in the serum was high. Cycle threshold value was 23, and the patient was put on remdesivir. She also received betamethasone 12 mg once daily for maturation of the fetal lungs.

The blood cultures were negative, and the C-reactive protein (CRP) was low (32 mg/L); broad-spectrum antibiotics (piperacillin/tazobactam) were commenced in case the coagulopathy was secondary to sepsis.

CTG raised suspicion of placental insufficiency due to fetal tachycardia. Fetal ultrasound showed moderate intrauterine growth retardation in both twins. T1 had umbilical artery Doppler flow type II (BFC 2) indicating an increased risk of placental insufficiency, and twin 2 (T2) had a normal umbilical blood flow (BFC 0). Other ultrasound measurements including blood flow in ductus venosus, uterine arteries, amniotic fluid levels, and fetal movements were normal.

Two hours after the ultrasound examination, CTG registration revealed a sudden bradycardia of T1 that prompted emergency caesarean section (CS). Fibrinogen concentrate (4 g), tranexamic acid (2 g), and FVIII concentrate (containing vWF, Haemate® 1000 U) were promptly administered, and magnesium sulphate was given intravenously for fetal neuroprotection.

CS was performed in general anesthesia, and T1 was delivered with Apgar 0, 0, 0 (weight 670 g) and T2 with Apgar 3, 8, 8 (weight 634 g). Resuscitation on T1 was unsuccessful, and T1 died. T2 was admitted to the neonatal intensive care unit and showed increased bleeding tendency. APTT (64 s) and INR (2.6) were prolonged, whereas fibrinogen was normal (2.2 g/L) during the first day and T2 developed an intracerebellar hemorrhage grade 2. The baby was treated with fresh frozen plasma and vitamin K and recovered.

The CS was uncomplicated (bleeding 300 mL). Within 12 hours, a vaginal bleeding of 600 mL was noted and the patient received two units of red blood cells due to low hemoglobin level (Hb 76 g/L) and 5 g of fibrinogen concentrate due to fibrinogen 1.3 g/L. No additional FVIII/vWF concentrate was used since the FVIII level was 1.05 kIU/L following antepartal substitution and delivery, which also ruled out antibodies against FVIII.

A few hours after the operation, the patient was treated with monoclonal antibodies (casirivimab/imdevimab).

The day after CS, FVIII level was increased (1.68 kIU/L) and fibrinogen level was stable (2.2 g/L). The vaginal bleedings following delivery were not deemed to be abnormal, and thromboprophylaxis was restarted (dalteparin 2500 U twice daily and after three days 5000 U twice daily). Screening for antiphospholipid and antinuclear antibodies was negative.

The patient had continuous treatment with remdesivir. Despite clinical improvement and lower CRP, she continued to exhibit some laboratory signs of overt inflammation, such as extreme reactive thrombocytosis, reaching its highest level (1494 × 10^9^/L) three days after the delivery, but not remarkably high ferritin (199 *μ*g/L). The liver enzymes normalized after delivery, and the kidney function continued to be normal.

The examination of the placentas showed no signs of thrombosis but widespread intervillositis and perivillous fibrinoid deposition in 90% of placental tissue, and both placentas were positive for COVID-19. The autopsy of T1 revealed merely a unilateral cleft lip palate, and PCR COVID-19 was negative in the lungs and liver. T2 had negative COVID-19 PCR in the blood and nasopharyngeal swab.

## 3. Discussion

This report illustrates a case of antenatal severe coagulopathy, which was assessed as a direct effect of a moderate COVID-19 infection with a severe placental insufficiency since it did not fulfill the criteria for preeclampsia or sepsis.

The patient was managed by a multidisciplinary team consisting of obstetric, coagulation, infectious disease, and anesthesiologic specialists. Differential diagnoses included COVID-19-associated coagulopathy, sepsis-induced coagulopathy, hemophilia carrier, acquired hemophilia, disseminated intravascular coagulation (DIC), and HELLP syndrome. The patient's laboratory picture consisted of abnormal coagulation and biochemistry tests, which however, were not typical of any of the differential diagnoses, whereas clinical symptoms were moderate.

COVID-19 coagulopathy is associated with thromboinflammation, comprising usually very high D-dimer, increased fibrinogen, increased or normal platelet counts, normal or prolonged PT-INR, prolonged CT, and short CFT as well as increased levels of FVIII and vWF [5, 6]. Thus, the low levels of fibrinogen and FVIII as well as the prolonged CFT and low MCF in our case were unexpected. Thromboinflammation with severe coagulopathy is usually associated with severe pulmonary manifestations, which was absent in our patient. She, however, developed a severe placental dysfunction with intrauterine fetal distress and perinatal death. The placenta showed signs of massive COVID-19 infection as well as intervillositis and perivillous fibrinoid deposition. These placental features have been reported in several cases with COVID-19 infection [7–10]. Data on COVID-19 coagulopathy during pregnancy is still scarce, and whether or how coagulopathy is associated with the placental changes is not clarified. In two previously published cases where HELLP syndrome was suspected, progressive thrombocytopenia, hypofibrinogenemia, and high D-dimer improved within two days after the delivery [11]. The low fibrinogen [11] could be attributed to postpartum hemorrhage (PPH). In our case, the fibrinogen started to decrease two days before delivery, and the patient did not have abnormal bleeding. A brief report from the ISTH SSC Subcommittee on Women's Health Issues in Thrombosis and Haemostasis showed that COVID-19-associated coagulopathy was present in 1% of the pregnancies, mainly in patients with severe disease. Elevated D-dimer and thrombocytopenia were the most common features [12].

## 4. Conclusion

Our report adds to the increasing knowledge of COVID-19 coagulopathy in pregnant women, but it also highlights the need for multidisciplinary management and broad differential diagnosis. It underlines that severe and persistent coagulopathy can occur even in patients without other signs of severe infection and may be associated with adverse obstetrical outcomes. Prompt management can minimize the risk for bleeding and thrombosis. Coagulopathy in pregnant women with COVID-19 warrants further studies.

## Figures and Tables

**Table 1:** Clinical laboratory data variable.

	Day 1	Day 2	Day 3 antepartum^∗^	Postpartum	Day 4	Day 5	Day 6	Reference ranges
CRP (mg/L)	42	34	37		25		39	<3
TPK (×10^9^/L)	285	210	164	157	165	190	1494	165-387
LD (microkat/L)	3.7	9.5	13.0	10.2	8.2	6.8	6.1	<3.5
Fibrinogen (g/L)	4.5	1.5	0,7	1.3	2.2	2.5	5.6	2.0-4.2
D-Dimer (mg/L)	3.7	>35	>35			0.53	1.47	<0,50
Antitrombin (kIE/L)	1.21	1.01	1.0		0.98	1.02	1.08	0.8-1.2
aPTT (sec)	30	54	56	39	39	31	28	20-30
PT-INR	0.9	0.9	0.9	1,0	1.0	0.9	0.9	<1.3
Factor VIII (kIE/L)			0.37	1.05	1.68	>1.8	>1.8	0.50-1.80
von Willebrand GP1bA (kIE/L)			>2.0	>2.0				0.50-1.90
INTEM CT (sec)			407	274				100-240
INTEM CFT (sec)			253	109				30-110
HEPTEM CT (sec)			295	222				100-240
HEPTEM CFT (sec)			222	120				30-110
EXTEM CT (sec)			121	82				38-79
EXTEM CFT (sec)			216	125				34-159
FIBTEM MCF (mm)			4	7				9-25

^∗^Prior to treatment with fibrinogen, factor VIII/vWF concentrate, and tranexamic acid.

## Data Availability

All data are available in the patient's record.
